# On the benefits of systematic reviews for wildlife parasitology

**DOI:** 10.1016/j.ijppaw.2016.05.002

**Published:** 2016-05-26

**Authors:** Neal R. Haddaway, Maggie J. Watson

**Affiliations:** a MISTRA EviEM, Stockholm Environment Institute, Box 24218, 104 51 Stockholm, Sweden; b Institute for Land Water and Society, Charles Sturt University, Albury, NSW 2640, Australia

**Keywords:** Systematic review, Literature review, Meta-analysis, Vote-counting, Comprehensiveness, Reliability, Parasitology, Wildlife

## Abstract

Systematic reviews and meta-analyses are widely accepted as the best means to synthesise quantitative or qualitative scientific evidence. Many scientific fields have embraced these more rigorous review techniques as a means to bring together large and complex bodies of literature and their data. Unfortunately, due to perceived difficulties and unfamiliarity with processes, other fields are not using these options to review their literature. One way to provide guidance for a specific field is to examine critically recent reviews and meta-analyses and to explain the advantages and disadvantages of the various review techniques. In this paper, we examine review papers in the emerging field of wildlife parasitology and compare five different literature review types—configurative narrative review, aggregative scoping review, aggregative literature review, aggregative meta-analysis, and aggregative systematic review. We found that most literature reviews did not adequately explain the methodology used to find the literature under review. We also found that most literature reviews were not comprehensive nor did they critically appraise the literature under review. Such a lack severely reduces the reliability of the reviews. We encourage all authors to consider using systematic reviews in the future, and for authors and peer-reviewers to be aware of the limitations of non-systematic reviews.

## Highlights

• Systematic reviews aim to increase comprehensiveness, objectivity and transparency.
• Recent wildlife parasitology reviews fail to use systematic methods to avoid bias.
• We highlight what should be done to mitigate bias and increase reliability.

## Graphical abstract

**Figure:**
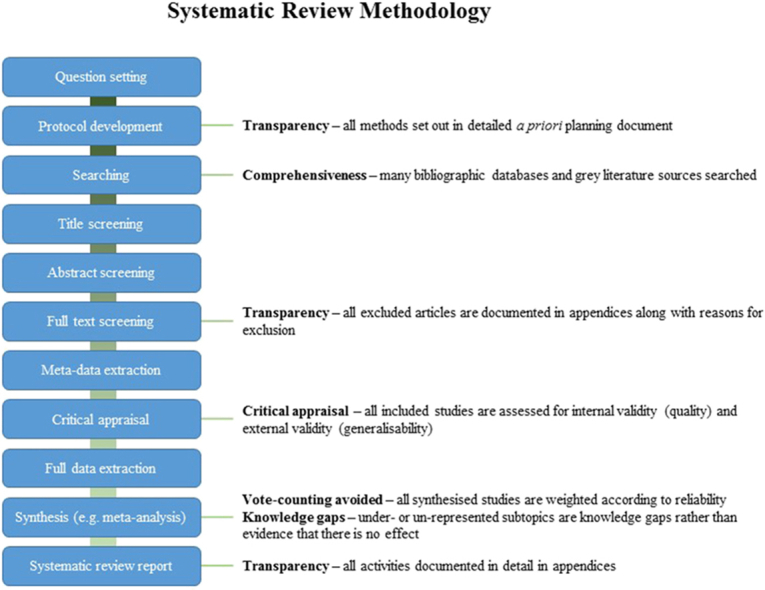

## 1 Introduction

Literature reviews provide vital means of synthesising large bodies of evidence, and their importance becomes clear considering the ever-increasing rate of research publication (Pautasso, 2012). In addition to acting as bibliographies of relevant research, reviews can estimate effect sizes of particular interventions or treatments (i.e. via meta-analysis), and can also examine the impact of context (i.e. heterogeneity) (Koricheva et al., 2013). Systematic reviews are a specific subset of literature reviews that aim to employ strict methods when searching for, screening, critically appraising and synthesising studies to maximise reliability through transparency, repeatability and objectivity (Higgins and Green, 2011). While systematic reviews can be resource-intensive, traditional literature reviews (clinical reviews and vote-counting reviews) can adopt systematic approaches to improve their reliability with minimal additional effort (Haddaway et al., 2015). Traditional reviews that do not adopt such approaches are susceptible to a number of limitations, including selection bias and publication bias that can reduce the reliability of the review outputs. All literature reviews (including systematic reviews) vary in their reliability, but systematic approaches can help to reduce susceptibility to a number of different biases.

Here, we outline the limitations associated with traditional literature reviews and what can be done to mitigate them using systematic review methodology. We illustrate our argument using recently published reviews in the wildlife-parasitology literature to provide examples of reviews that are at risk of unreliability and those that have succeeded in their stated aims. Wildlife parasitology is an ideal field to examine the variety of literature review approaches because it combines two disparate fields—ecology and parasitology. Ecologists have only recently abandoned the less formalised narrative review for systematic reviews and meta-analyses (Lortie, 2014), while parasitologists have a long tradition of following the Cochrane Collaboration methods of systematic reviews (Cook et al., 1997) because of the clinical nature of their work. Wildlife parasitology, with its roots both in ecology and veterinary medicine, has examples of all types of reviews in the recent literature and thus allows for a balanced examination of the pros and cons of each system.

## 2 Materials and methods

To identify review articles for this paper, we used systematic review methods to search for, screen and appraise reviews in the field of wildlife parasitology. We searched Web of Science Core Collections (Stockholm University subscription) on 18th April 2016 using the search string “wildlife AND parasit* AND (review OR “meta-analysis” OR metaanalysis)” in a Topic Words search. We restricted our search to the period from 2010 to 2015 due to resource limitations. We also selected a suite of academic parasitology journals that publish wildlife articles (Trends in Parasitology, International Journal for Parasitology – Parasites and Wildlife (IJP-PAW), Parasites and Vector, and Parasites and Vectors), and hand-searched within these journals for review papers published during the same period. Each journal was searched using its own search engine using the keyword ‘wildlife’, then identifying those articles that were categorised as ‘reviews’. The obtained search results from database and hand searches were then screened using the following inclusion criteria: i) they were a literature review; 2) their focus was on wildlife parasitology. We defined literature reviews as those that synthesise a data set for the specific purpose of detecting a pattern or trend. We have categorised reviews using an adapted version of the system set out by O’Connor and Sargeant (2015), as follows: 1) configurative narrative integrative reviews; 2) aggregative scoping reviews; 3) aggregative full literature review; 4) aggregative meta-analysis; and 5) aggregative systematic review (see Table 1). We have used two additional categories to the original system proposed by O’Connor and Sargeant (2015): configurative and aggregative, according to (Gough et al., 2015), with configurative reviews being model-forming, whilst aggregative reviews aim to collate and summarise study findings. In addition, we described five domains relating to the reliability and quality of the reviews, as follows: transparency, comprehensiveness, presence of vote-counting analysis, presence of critical appraisal of included studies, and confusion of no evidence of effect with evidence of no effect. These domains are described in detail below.

### 2.1 A lack of transparency

When reviewers do not describe how they searched for evidence, nor how they screened studies for inclusion, the review is then neither truly repeatable nor verifiable, as all science should be. By detailing searching and screening strategies, including the search strings and databases used, (making use of supplementary information) the work can be verified, repeated or updated (e.g. Bernes et al., 2015). In the fields of social science, human medicine and environmental management, systematic reviews are typically published with coordinating organisations that set standards in systematic review methods (such as the Collaboration for Environmental Evidence and the Cochrane Collaboration), which ensures that the review has been performed according to a high standard, typically involving a high level of transparency, but also ensuring the following risks to reliability are avoided.

### 2.2 A lack of comprehensiveness

Reviewers can (knowingly or unknowingly) introduce bias by selecting evidence to include in a review (selection bias), since it is impossible to ascertain whether the studies included are representative of the whole body of evidence. This can be particularly problematic if included studies originate from a research group with one school-of-thought (research bias, Gurevitch et al., 2000). A lack of comprehensiveness can also occur if publication bias (the tendency for significant research to be more frequently published than non-significant research) is not explicitly dealt with (i.e. by searching for grey literature) or discussed. Publication bias is a common problem with meta-analyses (Sutton et al., 2000), resulting in a potentially unreliable but precise result that can adversely affect decision-making. Reliable reviews should aim to collate all available evidence on a topic by using predefined search strategies in multiple academic databases and by searching for grey literature where appropriate (Haddaway and Bayliss, 2015).

### 2.3 Vote-counting

Meta-analysis combines results from multiple studies, increasing statistical power and allowing for investigation of sources of heterogeneity (Koricheva et al., 2013), and considers the magnitude and variability of effects rather than just the direction. Vote-counting (categorising studies as positive, negative or non-significant), however, ignores magnitude and variability. Vote-counting makes it impossible to examine non-significant trends that are only seen to be significant when assessed at a sufficient level of replication across multiple studies, and it also treats all studies as having the same level of reliability. Vote-counting should, therefore, always be avoided. Instead, meta-analysis that includes critical appraisal (see below) should be performed where possible (but see Lafferty (1997) for an alternate method of effect size presentation). Alternatively, studies should be considered as a whole, without tallying results based on their individual statistical significance, for example through a narrative that describes overall trends using tables and figures without focusing on the frequency of significance.

### 2.4 A lack of critical appraisal

Critical appraisal, the careful assessment of individual studies based on their reliability or susceptibility to bias, is a vital step in reviewing evidence (Higgins and Green, 2011), since even academic, peer-reviewed literature can be unreliable or wrong (Bohannon, 2013), and since research will vary in its relevance to any review. Without appraisal of the reliability of included research, for example, an unreplicated study would be given the same weight as a highly replicated randomised control trial. Some studies are inherently more accurate (e.g. due to sample size), some are more valid (e.g. due to control matching), and some are more biologically relevant (e.g. truly different due to underlying genetics, taxonomy or ecology). All reviews should assess validity, and weight or exclude evidence accordingly when synthesising it (see, e.g. Higgins and Green, 2011). For example, in a systematic review on the effectiveness of biomanipulation as a means for reducing eutrophication in inland waters, Bernes et al. (2015) critically assess the internal validity (quality) and external validity (generalisability) of each included study in their review and meta-analysis, excluding studies that failed to report baseline conditions or the intensity of the intervention in sufficient detail.

### 2.5 No evidence of effect ≠ evidence of no effect

A lack of evidence does not equate to evidence of no effect. A significant body of reliable evidence demonstrating non-significant effects does provide an evidence of no effect, but this conclusion should be based on a comprehensive search and reviewers should not confuse no evidence of effect with evidence of no effect. A trend towards more positive research is an expected result of publication bias that plagues academic publishing. For example, reviewers should perform critical appraisal, have a sufficient evidence base on which to base their conclusions, and be very cautious of interpreting a lack of studies as a lack of effect.

And finally, as an example of how a lack of transparency, comprehensiveness and critical appraisal and the use of vote-counting can severely limit the usefulness of a review, one paper, Pedersen and Fenton (2015) is examined in depth.

## 3 Results

Our search of Web of Science Core Collections returned 76 results for the period of 2010–2015 (132 for the entire date range of the database). A total of 22 studies were included after screening of abstracts. These articles were combined with 15 studies identified through hand searching of selected journals, leaving 37 relevant reviews. These reviews are described in detail in Table 2.

**Fig. 1:**
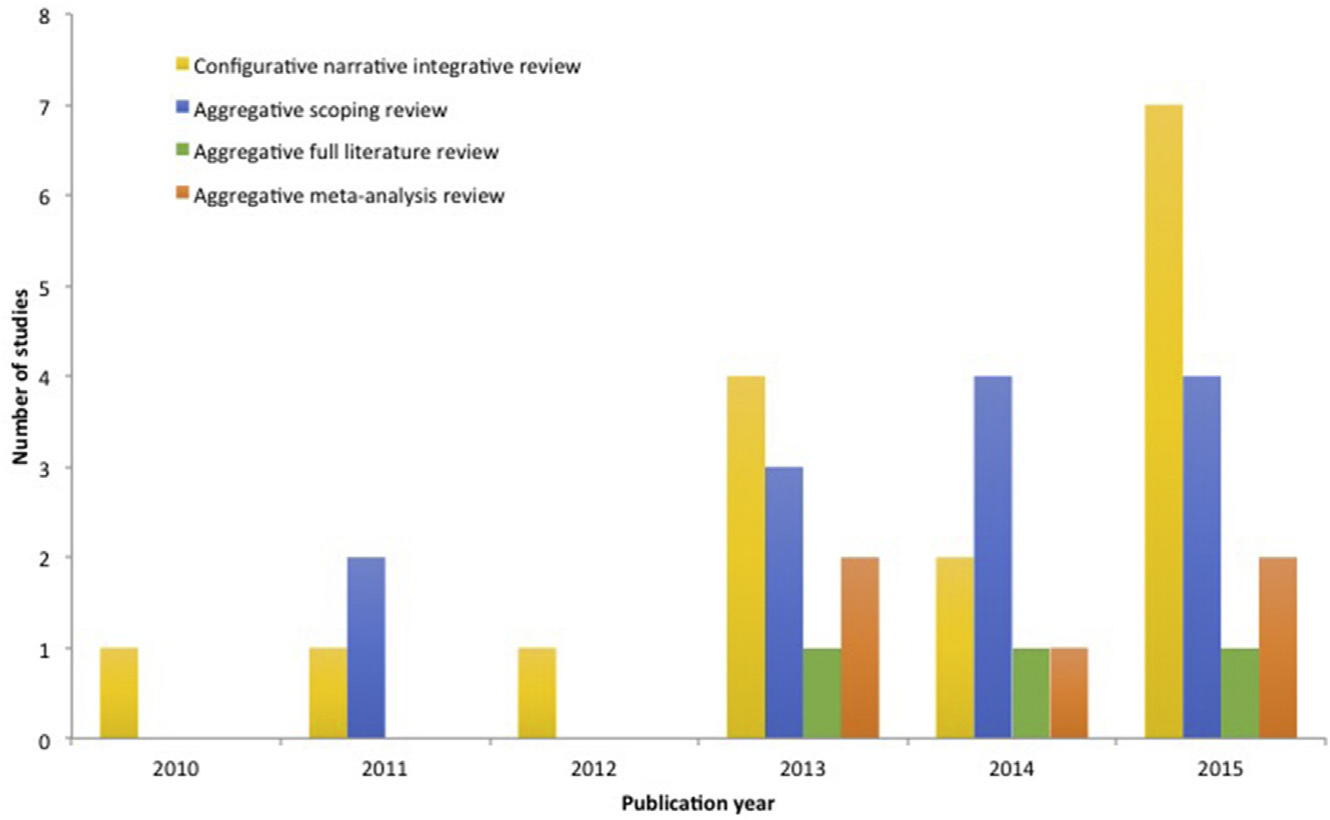
Review types identified in the field of wildlife parasitology from 2010 to 2015.

According to our schema we identified 16 configurative narrative integrative reviews, 13 aggregative scoping reviews, 3 aggregative full literature reviews, 5 aggregative meta-analyses, and no aggregative systematic reviews. The number of reviews in total has increased over time as is expected given current trends in publishing (Fig. 1).

The configurative reviews typically lacked transparency that would make it impossible to repeat the searches, although two reviews provided some details of searches (Carmena and Cardona, 2013, Medlock et al., 2013). As a result, it is not possible to assess comprehensiveness for these reviews. However, the nature of configurative reviews does not necessarily require comprehensiveness, since information saturation when building conceptual models means that at a certain point the addition of further studies would not add information to the conceptual model (Gough et al., 2015). Typically these types of reviews do not set out to synthesise study findings, and so vote-counting only occurred in one review (Alasaad et al., 2013).

Of greater interest to an assessment of the quality of reviews in wildlife parasitology is a validity of aggregative reviews that aim to collate study findings. Of the 13 scoping reviews, 10 provided some details of the search strategy, although most lacked sufficient detail to repeat the searches. Three reviews included vote-counting (Brearley et al., 2013, Fagre et al., 2015, Pedersen and Fenton, 2015). These reviews weighted all included studies equally and failed to account for either the size of the studies or the magnitude of study effects.

The aggregative full literature reviews provided some details of search strings used, but typically failed to provide details or results regarding the screening process for inclusion/exclusion of studies. Only one review attempts some assessment of the reliability of included studies (Tompkins et al., 2015).

Of the aggregative meta-analyses, all provided details of the search that was conducted, although one study failed to state when the searches were undertaken (Young et al., 2013). All papers included at least a basic assessment of bias. In some cases (e.g. Becker et al., 2015) this extended only to a discussion of low sample size precluding inclusion in meta-analysis, whilst other studies performed comprehensive assessments of possible bias, such as trim and fill statistics (Poulin & Kamiya, 2015).

In the single paper that was chosen for an extended analysis, Pedersen and Fenton 2015, as no details of search strategy or inclusion criteria were provided, a systematic search resulted in an additional thirty-one papers missing from the published literature review (see Supplement file). Vote-counting is performed and the authors discuss an apparent lack of non-significant/negative evidence across insect-specific treatments where only a small number of single studies show positive effects. No weighting is given to studies based on their internal or external validity. The authors go as far as to describe the type of study that is needed in an ideal situation but make no attempt to appraise critically the included studies against this standard. A very small number of studies showing non-significant results is described as evidence of no effect rather than a lack of evidence: “not all drugs were efficacious for treating wildlife parasites”. This may be a result of limitations in search method or of the studies (too small, poor methods, insufficient dose).

## 4 Discussion

We found that literature reviews in wildlife parasitology are mostly still using limited methodologies and techniques such as clinical and vote-counting reviews. Surprisingly we failed to find any reviews that would conform to the standards of the many systematic review coordinating bodies in the broad fields of social science, medicine and environmental management. Wildlife parasitology is a growing field emerging from a veterinary/medical basis into a more ecologically-minded discipline. We found that reliance on clinical and vote-counting reviews severely limits the reliability of a review. All forms of literature review are susceptible to biases that can affect their reliability, as discussed above.

Comparisons of various types of literature review show many differences in transparency, comprehensiveness, analysis type and evidence for no effect between traditional reviews and systematic reviews (Haddaway et al., 2015). The example papers used in this comparison of literature reviews come from different aspects of wildlife parasitology, but indicate how a move towards high quality reviews with systematic methodological approaches will benefit future research and decision-making. When writing a review, authors should remember that the readership may include those from other disciplines, and so clarity and transparency should be used as tools to maximise the review’s utility. List-style or clinical reviews are the norm in many fields of medical parasitology, but as these reviews become more common in the field of ecology it is important to inform the reader about the methodology behind such reviews; including search terms, data bases searched, and previous reviews sourced. Reviewers must also understand the limitations of vote-counting and, most importantly, the possibility that unreliable or inaccurate conclusions may be reached if the literature search is not systematic. In summary, we urge authors and peer-reviewers to adopt systematic approaches to literature reviews, and to consider using formalised systematic review methodology (see www.environmentalevidence.org or www.cochrane.org) where possible. In addition, we urge editors and journals to facilitate the publication of high quality, reliable systematic reviews but accepting systematic reviews as specific article types and by adopting standards for conduct and reporting of systematic reviews from other disciplines such as Preferred Reporting Items for Systematic Reviews and Meta-Analyses (PRISMA) for reporting (Moher et al., 2009) and the Methodological Expectations of Cochrane Intervention Reviews (MECIR) for reporting and conduct (http://editorial-unit.cochrane.org/mecir). Such standards are already adopted by other leading journals outside specialist fields, including PLOS ONE. Traditional reviews should particularly strive to be systematic where assessments of effectiveness are undertaken. Transparency, comprehensiveness and critical appraisal are central tenets of systematic review approaches and will yield the most reliable evidence. Lessons should be taken from systematic review guidance (Haddaway et al., 2015) to minimise bias and mitigate the limitations associated with non-systematic literature review methods.

## Figures and Tables

**Table 1:** Categories of review used for classifying literature reviews identified through this study.

Label	Description	Search	Inclusion	Appraisal	Synthesis	Analysis
Configurative narrative integrative review	Preliminary assessment of literature with the aim of introducing and interpreting an area of work	None identified	No details of inclusion/exclusion criteria	No or little quality assessment	Tabular with narrative commentary	Characterises literature by qualitative metric
Aggregative scoping review	Preliminary assessment of literature with the aim of identifying nature and extent of research	Some	No details of inclusion/exclusion criteria	No or little quality assessment	Tabular with narrative commentary	Characterises literature by some qualitative or quantitative metric often by vote-counting
Aggregative full literature review	Systematic search, appraisal and synthesis of research evidence to produce a best evidence synthesis	Exhaustive	No details of inclusion/exclusion criteria	Some quality assessment	Tabular with narrative commentary	Characterises literature by some qualitative or quantitative metric often by categories
Aggregative meta-analysis review	Systematic search with a statistical analytical component that combines the results to understand the effects	Exhaustive	No details of inclusion/exclusion criteria	Some quality assessment	Graphical, tabular, narrative commentary	Characterises literature by meta-analytical quantitative methods
Aggregative systematic review	Systematic search and inclusion stages with an assessment of study liability	Exhaustive, including grey literature	Full details of inclusion/exclusion criteria	All studies included appraised for quality	Graphical, tabular, narrative	Characterises literature by some qualitative or quantitative synthesis

**Table 2:** Description and coding of reviews identified in this study. See Table 1 for explanation of review types.

Citation	Type of review	Transparency	Comprehensiveness	Analysis: none, vote-counting or effect sizes	Critical appraisal	No evidence of effect vs. evidence of no effect
Otranto et al. (2015)	Configurative narrative integrative review	None	Unknown	None	None	None
Scasta (2015)	Configurative narrative integrative review	None	Unknown	None	None	None
Fagre et al. (2015)	Aggregative scoping review	Systematic search with search strings, dates, no search of grey literature and no justification for its exclusion	Unknown	Vote-counting (presence absence)	None	Explicit indication that the absence of effects does not mean that pathogens are not having an effect, merely that documentation does not exist for wild populations
Wiethoelter et al. (2015)	Aggregative scoping review	Systematic search with search strings, dates, no search of grey literature and no justification for its exclusion	Unknown	Temporal trends	None	Extended discussion on the fact this review was not intended to look for evidence of effects, just scientific interest in the topic
Becker et al. (2015)	Aggregative meta-analysis review	Systematic search with search strings, dates, no search of grey literature and no justification for its exclusion	Unknown	Effect sizes	Sample sizes mentioned with some analyses not possible due to low sample size, correction for bias	Evidence of no effect visualised in graphical form
Souza et al. (2015)	Configurative narrative integrative review	None	Unknown	None	None	None
Messenger et al. (2015)	Aggregative scoping review	Systematic search with search strings, dates, no search of grey literature and no justification for its exclusion	Unknown	Temporal trends	None	None
Fuehrer (2014)	Aggregative scoping review	Systematic search with search strings, no dates, no search of grey literature and no justification for its exclusion	Unknown	None	None	None
Civitello et al. (2014)	Aggregative meta-analysis review	Systematic search with search strings, dates, no search of grey literature and no justification for its exclusion	Unknown	Effect sizes	Data were known to be nonindependent, therefore no funnel plots or rank correlation tests to assess publication bias	None
Rowley et al. (2013)	Aggregative scoping review	None	Unknown	None	None	None
Carmena and Cardona (2013)	Configurative narrative integrative review	Databases searched are stated along with date ranges, but no search string and no screening methods	Unknown	None	Caution regarding sample size of some studies	None
Joseph et al. (2013)	Aggregative full literature review	Systematic search with search strings, dates, and search of grey literature	Unknown	description of trends	None	None
Young et al. (2013)	Aggregative meta-analysis review	Databases searched are stated along with search string but no dates (except for results) and no screening methods or grey literature search nor reason why not searched	Unknown	Effect sizes	trim and fill and forest plots	Strong evidence was not found which doesn’t mean it isn’t there, just this study didn’t find it
Brearley et al. (2013)	Aggregative scoping review	Databases searched are stated along with search string, but no date ranges search and little information on screening methods	Unknown	Vote-counting	Caution regarding number of studies used to infer trends	No mention of “no effect”
Mukaratirwa et al. (2013)	Configurative narrative integrative review	None	Unknown	None	None	None
Strauss et al. (2012)	Configurative narrative integrative review	None	Unknown	None	None	None
Jenkins et al. (2011)	Configurative narrative integrative review	None	Unknown	None	None	None
Wendte et al. (2011)	Aggregative scoping review	Databases searched are stated along with search string, but no date ranges and little information on screening methods	Unknown	None	None	None
Tompkins et al. (2011)	Aggregative scoping review	None	Unknown	None	None	None
Power (2010)	Configurative narrative integrative review	None	Unknown	None	None	None
Alasaad et al. (2013)	Configurative narrative integrative review	None	Unknown	Vote-counting	None	None
Fuehrer (2014)	Aggregative scoping review	Systematic search with search strings, no dates, no search of grey literature and no justification for its exclusion	Unknown	None	None	None
Donahoe et al. (2015)	Configurative narrative integrative review	None	Unknown	None	None	None
Duron et al. (2015)	Configurative narrative integrative review	None	Unknown	None	None	None
Pedersen and Fenton (2015)	Aggregative scoping review	None	See Supp Info for examples of papers missed	Vote-counting	None	A very small number of studies showing non-significant results is described as evidence of no effect rather than a lack of evidence: “not all drugs were efficacious for treating wildlife parasites”. This may be a result of limitations in search method or of the studies (too small, poor methods, insufficient dose)
Tompkins (2015)	Aggregative full literature review	Details of search strings used and databases searched, however no details of number of relevant publications identified through searching or screening, however no search of the grey literature and no justification for its exclusion	Unknown	A list of studies that fit the parameters of the search are provided, but the authors do not attempt to determine significance of evidence, only categorical classification of possible drivers of trends observed	Some	Explicitly discussed and the authors suggest that a more powerful analysis would be possible if there were more studies to examine
Poulin and Kamiya (2015)	Aggregative meta-analysis review	Details of search strings used, databases searched, details of number of publications and exclusion criteria however no search of the grey literature and no justification for its exclusion	Unknown	Effect sizes	Yes	Explicitly discussed under heterogeneity
Dutari and Loaiza (2014)	Aggregative scoping review	Few details of search strategy	Unknown	Temporal trends	None	None
Medlock et al. (2013)	Configurative narrative integrative review	Details of search strings used and databases searched, however no details of number of relevant publications identified through searching or screening, no search of the grey literature and no justification for its exclusion	Unknown	None	None	None
Walker and Morgan (2014)	Aggregative scoping review	Details of databases used, and search strings for some but not others, some other information	Unknown	Temporal trends	None	None
Watson (2013)	Aggregative meta-analysis review	Details of search strings used, databases searched, details of number of publications and exclusion criteria however no search of the grey literature and no justification for its exclusion	See Supp Info for examples of papers missed	Effect sizes	Sample sizes and species bias	Explicit discussion of file drawer problem
Catalano et al. (2014)	Configurative narrative integrative review	None	Unknown	None	None	None
Walker et al. (2014)	Aggregative full literature review	Details of search strings used and databases searched, however no details of number of relevant publications identified through searching or screening, however no search of the grey literature and no justification for its exclusion	Unknown	temporal trends and liability analysis	None	None
Shamsi (2014)	Aggregative scoping review	Some details of search string and dates	Unknown	temporal trends and liability analysis	None	None
Lymbery et al. (2014)	Configurative narrative integrative review	None	Unknown	percents	explicit discussion of bias due to selection process of studies used in analysis	None

## Appendix A Supplementary data

Relevant studies omitted from Pedersen and Fenton (2015).
