# Supplementary material for: On the benefits of systematic reviews for wildlife parasitology
Source: Int J Parasitol Parasites Wildl. 2016 May 26;5(2):184–91. doi: 10.1016/j.ijppaw.2016.05.002 (PMC5005428; doi:10.1016/j.ijppaw.2016.05.002)
Supplement: Supplementary file 1 — Relevant studies omitted from Pedersen and Fenton (2015). [file mmc1.docx]

Table S1 Antiparasite drug treatment studies on wildlife missing from Pedersen and Fenton [1] but included in [2] or [3]. An asterisk indicates additional studies found while compiling this table.

| Drug type | Drug name | | Target parasites | | | Host species | | Dose and/or administration | | Refs |
| --- | --- | --- | --- | --- | --- | --- | --- | --- | --- | --- |
| Anthelmenthic | Fenbendazole | | Mixed helminths | | | Common eider, *Somateria mollissima* | | 26 mg/kg, oral, single dose | | [4] |
|  |  |  | Mixed helminths | | | American coot, *Fulica americana* | | 50 mg/kg, oral, single dose | | [5]* |
|  |  |  | Mixed nematodes | | | Mixed bovids and cervids | | 7.5 mg/kg, repeated dose | | [6]* |
|  | Ivermectin | | Mixed nematodes and arthropods | | | Cape ground squirrel, *Xerus inauris* | | 0.1 mL, injection, repeated dose | | [7, 8] |
|  |  |  | Mixed nematodes | | | Mixed bovids and cervids | | 0.2 mg/kg, repeated dose | | [6]* |
|  | Levamisole, oxyelozanide, ivermectin | | Mixed helminths | | | Eurasian oystercatcher, *Haematopus ostralegus* | | Mixed oral, single dose | | [9] |
| Insecticide | Dibrom (dimethyl 1,2-dibromo-2,2-dichloroethylphosphate) | | Mixed ectoparasites | | | Cliff swallow, *Petrochelidon pyrrhonota* | | Sprayed nests, repeated dose; fumigation, single dose | | [10,11] |
|  |  |  | Martin mite, *Dermanyssus prognephilus* | | | Purple martin, *Progne subis* | | Sprayed nests, repeated dose | | [12,13] |
|  | Dichlorvos, 2,2-dichlorovinyl dimethyl phosphate | | Nest mite, *Pellonyssus reedi* | | | House finch, *Carpodacus mexicanus* | | Pest-strip placed in nest, single dose | | [14] |
|  | Bromocyclen | | Sand martin flea, *Ceratophyllus styx,* Sand martin tick, *Ixodes lividus,* hippoboscid fly, *Sterepterix hirundinis* | | | Sand martin, *Riparia riparia* | | 4.25g/100g, dusted on nests, repeated dose | | [15] |
|  | Ivermectin | | Mixed ectoparasites | | | Crested tern, *Thalasseus bergii* | | 0.8g/L, topical, repeated dose | | [16] |
|  | Selamectin | | Mixed ectoparasites | | | Mountian brushtail possum, *Trichosurus cunninghami* | | 6mg/kg, topical, repeated dose | | [17] |
|  | Fipronil | | Fleas, to indirectly remove *Yersinia pestis* | | | California ground squirrel, Spermophilus beecheyi | | Varied dose on bedding and 15mg/kg topical, single dose | | [18]* |
|  |  |  | Mixed ectoparasites | | | Cape Ground Squirrel, *Xerus inauris* | | 0.29%, topical | | [7,8] |
|  |  |  | Mixed ectoparasites | | | Mountain brushtail possum *Trichosurus cunninghami* | | Topical, repeated dose | | [17] |
|  | Pyrethrum | | Poultry red mite, *Dermanyssus gallinae* | | | Rock dove, *Columba livia* | | 1%, fumigation, repeated dose | | [19] |
|  | Pyrethrins (mixed with carbaryl and piperonyl butoxide) | | Mixed ectoparasites | | | Columbian ground squirrel, *Spermophilus columbianus* | | Topical, repeated dose | | [20] |
|  | Pyrethrin | | Tropical fowl mite, *Ornithonyssus bursa* | | | Barn swallow, *Hirundo rustica* | | 0.47% sprayed on nests, single dose | | [21] |
|  |  |  | Tick, *Ixodes lividus* | | | Sand martin, *Riparia riparia* | | 0.25% sprayed on nests, single dose | | [22] |
|  |  |  | House martin bug, *Oeciacus hirundinis* | | | House martin, *Delihon urbica* | | 0.91% sprayed on nests, single or repeated dose | | [23] |
|  | Permethrin (mixed with piperonil butoxide) | | Feather mites | | | Barn swallow, *Hirundo rustica* | | 0.17% sprayed on nests, single dose | | [24] |
|  |  |  | Mixed ectoparasites | | | Blue tit, *Cyanistes caeruleus* | | 0.5% sprayed on nests, single dose | | [25] |
|  | Flumethrin | | Tick, *Argas robertsi* | | | Cattle egret, *Bubulcus ibis* | | 10g/L, 10–30mL sprayed on nests, single dose | | [26] |
|  | Deltamethrin | | Northern fowl mite, *Dermanyssus hirundinis* | | | Pied flycatcher, *Ficedula hypoleuca* | | 0.028% sprayed on nests, single dose | | [27] |
|  | Imidacloprid | | Fleas, to indirectly remove *Yersinia pestis* | | | California ground squirrel, Spermophilus beecheyi | | Varied dose on bedding and 15mg/kg topical, single dose | | [18]* |
|  | Carbaryl (1-naphthyl N-methyl-carbamate) | | Mixed ectoparasites | | | European starling, *Sturnus vulgaris* | | Dusted nests, single dose | | [28] |
| Antiprotozoal | Primaquine | | Haemoproteus prognei | | | House martin Delichon urbica | | 0.01 mg, injection, single dose | | [29] |
|  |  |  | H. and Leucocytozoon majoris | | | Blue tit, *Cyanistes caeruleus* | | 0.01 mg, injection, single dose | | [30] |
| Antibiotic | Cephalosporine (ceftiofur sodium cephalosporine) | | Mixed bacterial species | | | Magellanic penguin | | 12.5 mg Injection, repeated dose | | [31] |
|  | |  | |  |  | |  | |  |  |
|  |  | |  | | |  | |  | |  |

1 Pedersen, A.B. and Fenton A. (2015) The role of antiparasite treatment experiments in assessing the impact of parasites on wildlife. Trends Parasitol. 31, 200–211.

2 Watson, M.J. (2012) What drives population-level effects of parasites? Meta-analysis meets life-history. Int. J. Parasitol. Parasites Wildl. 2, 190–196

3 Møller, A.P. et al. (2009) A meta-analysis of parasite virulence in nestling birds. Biol. Rev. 84, 567–588

4 Hanssen S.A. et al. (2003) Costs of parasites in common eiders: effects of antiparasite treatment. Oikos 100, 105–111

5 Amundson, C.L. and Arnold, T.W. (2010) Anthelminthics increase survival of American coot (*Fulica americana*) chicks. Auk 127, 653–659

6 Goossens, E. et al. (2006) Evaluation of three strategic parasite control programs in captive wild ruminants. J Zoo Wildlife Med 37, 20–26

7 Hillegass, M.A. et al. (2010). Parasite removal increases reproductive success in a social African ground squirrel. Behav. Ecol. 21, 696–700

8 Scantlebury, M. et al. (2007) Energetic costs of parasitism in the Cape ground squirrel *Xerus inauris*. Proc. R. Soc. Lond. 274, 2169–2177

9 Van Oers, K. et al. (2002) Anthelminthic treatment negatively affects chick survival in the Eurasian oystercatcher *Haematopus ostralegus*. Ibis 144, 509–517

10 Brown, C.R. et al. (1995) Ectoparasites reduce long-term survival of their avian host. Proc. R. Soc. Lond. 262, 313–319

11 Brown, C.R. and Brown, M.B. (2004) Group size and ectoparsitism affect daily survival probability in a colonial bird. Behav. Ecol. Sociobiol. 56, 498–511

12 Moss, W.W. (1966) The biological and systematic relationships of the martin mite, *Dermanyssus prognephilus* Ewing (Acari: Mesostigmata: Dermanyssidae). Unpublished PhD thesis, The University of Kansas

13 Moss, W.W. and Camin, J.H. (1970) Nest parasitism, productivity, and clutch size in purple martins. Science 168, 1000–1003

14 Stoehr, A.M. et al. (2000) Nest mites (*Pellonyssus reedi*) and the reproductive biology of the house finch (*Carpodacus mexicanus*). Can. J. Zool. 78, 2126–2133

15 Alves, M.A.S. (1997) Effects of ectoparasites on the sand martin *Riparia riparia* nestlings. Ibis 139, 494–496

16 Watson, M.J. (2012) Effects of parasites on the crested tern *Thalasseus bergii*. Unpublished PhD thesis, School of Animal and Veterinary Sciences, Charles Sturt University, Wagga Wagga

17 Hufschmid, J. (2008) An investigation into lumbo-sacral dermatitis (“rumpwear”) in mountain brushtail possums (*Trichosurus cunninghami*). Unpublished PhD thesis, Faculty of Veterinary Science, University of Melbourne

18 Metzger, M.E. and Rust, M.K. (2002) Laboratory evaluation of fipronil and imidacloprid topical insecticides for control of the plague vector *Oropsylla montana* (Siphonaptera: Ceratophyllidae) on California ground squirrels (Rodentia: Sciuridae). J. Med. Entomol. 39, 152–161

19 Clayton, D.H. and Tompkins, D.M. (1995) Comparative effects of mites and lice on the reproductive success of rock doves (*Columba livia*). Parasitology 110, 195–206

20 Neuhaus P. (2003) Parasite removal and its impact on litter size and body condition in Columbian ground squirrels (*Spermophilus columbianus*). Proc. R. Soc. Lond. 270, S213–S215

21 Møller, A.P. (1990) Effects of parasitism by a haematophagous mite on reproduction in the barn swallow. Ecology 71, 2345–2357

22 Szép, T. and Møller, A.P. (1999) Cost of parasitism and host immune defence in the sand martin *Riparia riparia*: a role for parent-offspring conflict? Oecologia 119, 9–15

23 de Lope, F. et al. (1998) Parasitism, immune response and reproductive success in the house martin *Delichon urbica*. Oecologia 114, 188–193

24 Pap, P.L. et al. (2005) Host-symbiont relationship and abundance of feather mites in relation to age and body condition of the barn swallow (*Hirundo rustica*): an experimental study. Can. J. Zool. 83, 1059–1066

25 Tomás, G. et al. (2007) Consequences of nest reuse for parasite burden and female health and condition in blue tits, *Cyanistes caeruleus*. Anim. Behav. 73, 805–814

26 McKilligan, N.G. (1996) Field experiments on the effect of ticks on breeding success and chick health of cattle egrets. Aust. J. Ecol. 21, 442–449

27 Bauchau, V. (1997) Do parasitic mites decrease growth of nestling pied flycatchers *Ficedula hypoleuca*? Ardea 85, 243–247

28 Fauth, P.T. et al. (1991) Ectoparasites and the role of green nesting material in the European starling. Oecologia 88, 22–29

29 Marzal, A. et al. (2005) Malarial parasites decrease reproductive success: an experimental study in a passerine bird. Oecologia 142, 541–545

30 Merino et al (2000) Are avian blood parasites pathogenic in the wild? A medication experiment in blue tits (*Parus caeruleus*). Proc. R. Soc. Lond. 267, 2507–2510

31 Potti, J. et al. (2002) Bacteria divert resources from growth for magellanic penguin chicks. Ecol. Lett. 5, 709–714
